# Role of bile acids in overweight and obese children and adolescents

**DOI:** 10.3389/fendo.2022.1011994

**Published:** 2022-11-30

**Authors:** Cosimo Giannini, Concetta Mastromauro, Serena Scapaticci, Cristina Gentile, Francesco Chiarelli

**Affiliations:** Department of Pediatrics, University of Chieti, Chieti, Italy

**Keywords:** bile acids, childhood obesity, overweight children, gut microbiota, metabolism

## Abstract

Bile acids (BAs) are amphipathic molecules synthetized in the liver. They are primarily involved in the digestion of nutrients. Apart from their role in dietary lipid absorption, BAs have progressively emerged as key regulators of systemic metabolism and inflammation. In the last decade, it became evident that BAs are particularly important for the regulation of glucose, lipid, and energy metabolism. Indeed, the interest in role of BA in metabolism homeostasis is further increased due to the global public health increase in obesity and related complications and a large number of research postulating that there is a close mutual relationship between BA and metabolic disorders. This strong relationship seems to derive from the role of BAs as signaling molecules involved in the regulation of a wide spectrum of metabolic pathways. These actions are mediated by different receptors, particularly nuclear farnesoid X receptor (FXR) and Takeda G protein coupled receptor 5 (TGR5), which are probably the major effectors of BA actions. These receptors activate transcriptional networks and signaling cascades controlling the expression and activity of genes involved in BA, lipid and carbohydrate metabolism, energy expenditure, and inflammation. The large correlation between BAs and metabolic disorders offers the possibility that modulation of BAs could be used as a therapeutic approach for the treatment of metabolic diseases, including obesity itself. The aim of this review is to describe the main physiological and metabolic actions of BA, focusing on its signaling pathways, which are important in the regulation of metabolism and might provide new BA -based treatments for metabolic diseases.

## 1 Introduction

Bile acids (BAs) are amphipathic molecules obtained from cholesterol in hepatocytes (1). Their best-known role consists of the digestion and absorption of dietary lipids, steroids, and lipophilic nutrients. In fact, after meal intake, they are released in the small intestine where they are organized in micelles with phospholipids and cholesterol, thus permitting the absorption of nutrients. A greater majority (about 95%) returns to the liver after being reabsorbed in the last portion of intestine, while the remainder (5%) is eliminated in stool (2). BA absorption is mediated by enterohepatic circulation. This mechanism is highly important in humans not only for nutrient absorption and for regulation of whole-body lipid metabolism, but also for preserving the entire metabolic homeostasis (3).

In the last decade, BAs have become increasingly important not only as a facilitator of nutrient digestion (4) but also in the entire metabolism, and in order to understand the complex metabolic role of BAs, a large number of research have been started (5). Reports have strongly suggested that there is a close mutual relationship between BA and metabolism (6). The interest in BA additional actions is further increased in recent years, concomitant to the global increase in obesity and related metabolic disorder prevalence. Indeed, the prevalence of obesity is constantly increasing from childhood with a consequent increased risk of several complications from this early age (7, 8). An emerging hypothesis postulates that BAs might be an important modulator of obesity itself and its related consequences.

The relationship between BAs and metabolism seems to derive from the role of BAs as signaling molecules involved in the regulation of a wide spectrum of metabolic pathways, including lipid and glucose metabolism (9). This regulatory role derives from the BA-dependent activation of intracellular ligand-activated nuclear receptors, the most important of which is represented by the farnesoid X receptor (FXR) and the G-protein-coupled BA receptor (GPCR) like TGR5. These receptors are probably the major effectors of BA actions. The activation of these signaling pathways might have a key role in the regulation of intestinal inflammation as well as in the improvement of insulin sensitivity and its related diseases (2, 3). In fact, different studies have shown that abnormalities of BA regulation are involved in impaired lipid, glucose, and energy metabolism that subsequently led to metabolic diseases like diabetes, non-alcoholic fatty liver disease, cardiovascular risk, and obesity.

The aim of this review is to describe the main physiological and pathological metabolism of BAs, focusing on its signaling pathways, which are important in the regulation of metabolism, specifically in insulin resistance and obesity. In addition, we discuss the development of new BA -based treatments for metabolic diseases.

## 2 Physiological roles of bile acids

### 2.1 Primary bile acid synthesis

**Figure 1:**
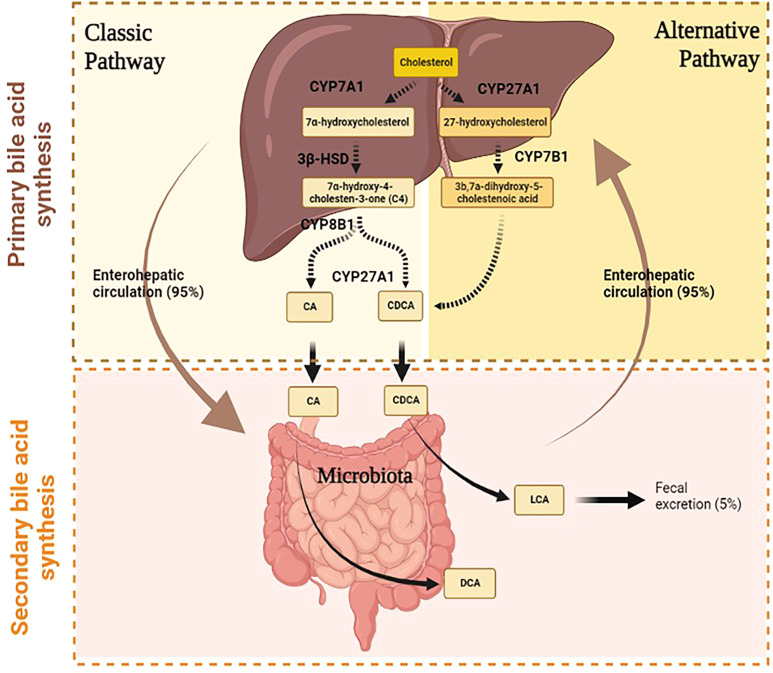
Primary and secondary bile acids synthesis in humans.

BAs are amphipathic molecules synthesized from cholesterol in the hepatocytes by primary BA synthesis. The daily cholesterol amount is utilized for the synthesis of different elements. The majority is employed for BA synthesis (50%), the remaining 40% is used for biliary secretion, and a small amount (10%) is needed for steroid hormone and membrane synthesis (10). The primary BA synthesis develops through two different pathways, namely, classical and alternative (**Figure 1**). They are responsible for primary BA production, namely, cholic acid (CA) and chenodeoxycholic acid (CDCA) (10).

#### 2.1.1 Classical pathway

The classical pathway starts in the endoplasmic reticulum of hepatocyte by the activity of microsomal cytochrome P450 cholesterol 7α-hydroxylase (*CYP7A1*) (2) (**Figure 1**). This enzyme catalyzes the production of 7α-hydroxycholesterol from cholesterol and consequently regulates the overall amount of BA production. In fact, CYP7A1 is considered the rate-limiting enzyme of BA synthesis, since it is regulated by negative feedback based on CA level (1). The relevance of this step is demonstrated in mice with CYP7A1 deficiency (11). Ishibashi and colleagues have shown that these mice had a high incidence of postnatal mortality due to liver failure and malabsorption (12). Indeed, Pullinger et al. have identified a *CYP7A1* frameshift mutation that blocks enzyme activity in three subjects with statin-resistant hypercholesterolemia and premature gallstone disorder (13).

The 7α-hydroxycholesterol is converted to 7α-hydroxy-4-cholesten-3-one (C4) by the action of 3β-hydroxy-Δ5-C27-hydroxysteroid dehydrogenase (*3β-HSD*), which catalyzes the hydroxylation of C4 at the C-12 position. The 7α-hydroxy-4-cholesten-3-one (C4) is a precursor of both CA and CDCA, thus determining another important step of this cascade, which establishes the rate of BA synthesis (14). Thereafter, mitochondrial cytochrome P450 27α-hydroxylase (*CYP27A1*) catalyzes the cleavage of the 3-carbon unit from the steroid side chain and C-24 BA s and propionyl-CoA are produced, leading to the synthesis of CA (10). This pathway represents the major BA biosynthetic pathway producing among 90% of the BA pool.

#### 2.1.2 Alternative pathway

Under physiological conditions, the alternative pathway contributes to only 10% of total synthesis of BA in humans, but it may be up regulated under pathological states such as liver disease (1). Instead, this pathway produces about 50% of BA composition in rodents. The alternative pathway starts with the activation of *CYP27A1*, which catalyzes the conversion of cholesterol to 27-hydroxycholesterol. The oxysterol 7a-hydroxylase (*CYP7B1*) converts the 27-hydroxycholesterol to 3b,7a-dihydroxy-5-cholestenoic acid, which then forms the primary BA s, namely, CA and CDCA in hepatocytes (2) (**Figure 1**).

#### 2.1.3 The enterohepatic circulation of bile acids

After the liver synthesis process, CA and CDCA are conjugated to glycine and taurine to form conjugated BAs (10). The conjugated BAs are then secreted into the bile canaliculi through the action of the bile salt export pump (BSEP) and are thus collected in the gallbladder. After meal intake, enteroendocrine I cells release the cholecystokinin (CCK), which leads to gallbladder contraction and the subsequent release of bile into the duodenum across the bile duct. In the intestinal tract, BAs are needed for the digestion and absorption of lipids since they activate pancreatic lipase and form micelles containing dietary fat facilitating the solubilization of fatty acids and lipophilic vitamins (A, D, E, and K) (15). In detail, these properties derive from the presence of hydroxyl groups in BAs, which make BAs amphipathic molecules since they present a hydrophilic side and a hydrophobic side that give them strong detergent properties. CA is more soluble than CDCA because it presents three hydroxyl (HO) groups compared to CDCA, which has only two hydroxyl groups. Most BAs, about 95% of total amount, are reabsorbed in the terminal ileum through an active process that requires the apical-sodium-dependent BA transporter (ASBT/SLC10A2). After BAs enter the enterocytes, they are then secreted at the basolateral membrane of enterocytes by the heterodimeric organic solute transporters α and β (OST α/β). BAs go back to the liver across portal veins, where they are internalized by transporters in the hepatocytes (NTCP, OAT, OATP, and mEH) (16, 17). This enterohepatic cycle of BA s is repeated from six to eight times a day. Within hepatocytes, free BAs are secreted with newly synthesized BAs into bile canaliculi; this mechanism is useful to balance fecal loss (18). In fact, the remaining 5% of the total BAs (approximately 0.5 g/day) is excreted into the feces and urine; this loss is thus replaced by the *de novo* synthesis in the hepatocytes.

### 2.2 Secondary bile acid synthesis

The BAs excreted into feces and urine are first metabolized by gut microbiota, which converts the primary BAs to secondary BAs (17), (16), (**Figure 1**). In detail, in the intestinal tract, a fraction of conjugated CA and CDCA are de-conjugated by gut bacterial bile salt hydroxylase (BSH) to form free BAs; then, bacterial 7α-dehydroxylase activity subtracts a 7-HO group from CA and CDCA to produce deoxycholic acid (DCA) and lithocholic acid (LCA), respectively (3). The DCA represents 20% of the BA pool together with CA and CDCA, which makes up the remaining 80% (2). On the other hand, since LCA is a toxic and insoluble BA, more of it is eliminated in stool and returns to the liver in small amounts where it is sulfo-conjugated for secretion into urine (10) (3),. Furthermore, the intestinal bacteria convert DCA and LCA into iso-DCA and iso-LCA (3β-OH epimers) through the iso-BA pathway. This modification reduces their bactericidal effects. The potential antimicrobial effect of BAs derives from the possibility of damaging bacterial membranes and altering intracellular macromolecular structures by using detergent properties (18).

## 3 Metabolic functions of bile acids

Apart from their role in dietary lipid absorption and homeostasis, BAs have progressively emerged as key regulators of systemic metabolism and inflammation (9), raising the possibility that modulation of BAs could be used as a therapeutic approach for the treatment of metabolic diseases, including obesity (19).

Although BAs can activate many signaling pathways, most of the metabolic effects of BAs are largely mediated by the nuclear receptor FXR and the G-protein-coupled receptor TGR5, which seem to be the major effectors of BA actions on regulation of glucose, lipid, and energy metabolism (20). Therefore, the characterization of these BA receptors and related pathways might pave the way for understanding BA function and future therapeutic opportunities. In **Table 1**, we have summarized the main BA receptors and their main metabolic effects.

**Table 1:** Bile acid sensors, targets, and metabolic effects.

Pathways	Target	Effects
**Bile acid metabolism**		
• FXRα–SHP • FXRα–FGF15–FGFR4 • JNK	• CYP7A1 • CYP8B1	Decreased bile acid synthesis
• FXRα	• BACS • BAT • UGT2B4 • SULT2A1	Increased bile acid conjugation
• FXRα–SHP • FXRα	• NTCP • ASBT • BSEP • MDR2 • MRP2 • I-BABP • OSTα, OSTβ	Modulation of bile acid enterohepatic cycling
**Lipid metabolism**		
• FXRα	• Bile acid synthesis	Increased serum LDL cholesterol
• FXRα	• SRB1 • APOA1 • PLTP	Decreased serum HDL cholesterol
• FXRα–SHP–SREBP1c	• ACC1, ACC2 • FAS • APOC3, APOC2 • LPL	Decreased serum TG
• FXRα	• VLDLR • SDC1	Decreased serum TG
**Energy homeostasis**		
• TGR5	• D2 • PGC1α	Increased energy expenditure
**Glucose metabolism**		
• FXRα	• PEPCK • G6Pase	Decreased gluconeogenesis
• FXRα • PI3K– AKT–GSK3β-GS	• G6Pase • GSK3β	Increased glycogenesis
• TGR5	• GLP1	Increased incretin release

APO, apolipoprotein; ACC, acetyl-CoA carboxylase; ASBT, apical sodium dependent bile acid transporter; BACS, bile acid-CoA synthetase; BAT, bile acid-CoA:amino acid N-acetyltransferase; BSEP, bile salt export pump; CYP7A1, cholesterol 7α-hydroxylase; CYP8B1, sterol 12α-hydroxylase; D2, type 2 iodothyronine deiodinase; FAS, fatty acid synthase; FGF15, fibroblast growth factor 15; FXR-α, farnesoid X receptor-α; G6Pase, glucose-6-phosphatase; GLP1, glucagon-like peptide 1; GS, glycogen synthase; GSK3β, glycogen synthase kinase 3β; HDL, high-density lipoprotein; I-BABP, ileal bile-acid-binding protein; JNK, c-Jun N-terminal kinase; LPL, lipoprotein lipase; MDR2, multidrug resistance protein 2; MRP2, multidrug resistance-associated protein 2; NTCP, sodium taurocholate cotransporting polypeptide; OST, organic solute transporter; PEPCK, phosphoenol pyruvate carboxykinase; PGC1-α, peroxisome proliferator-activated receptor-γ co-activator 1α; PI3K, phosphoinositol-3-kinase; PLTP, phospholipid transfer protein; SDC1, Syndecan-1; SHP, small heterodimer partner (also known as NR0B2); SRB1, scavenger receptor class B type I; SREBP1c, sterol response element binding protein 1c, TG, triglycerides; TGR5, also known as G-protein coupled bile acid receptor 1 (GPBAR1); SULT2A1, dehydroepiandrosterone sulfotransferase; UGT2B4, uridine 5-diphosphate glucuronosyltransferase 2B4; VLDL, very-low-density lipoprotein; VLDLR, VLDL receptor.

### 3.1 BA receptors and related pathways

#### 3.1.1 Nuclear receptor signaling pathways

Since the cloning of the first member of the family in 1985, many members of the nuclear receptor (NR) family have been discovered, comprising 48 members in the human genome today (21). Despite the great variability exhibited by the different members of the NR family, a common structure can be highlighted and consists of a ligand-independent transcriptional activation function (AF-1) domain (A/B), a core DNA-binding domain (C), a hinge region (D), a COOH-terminal ligand-binding domain (E), and a ligand-dependent activation function (AF-2) domain (F). Ligand binding to an NR determines a conformational change that facilitates the replacement of a corepressor with a coactivator, which allows the transcription of specific gene targets. Among NRs, the FXR, the pregnane X receptor (PXR), and the vitamin D receptor (VDR) are considered primary BA “sensors”, as they directly bind them. However, small heterodimer partner (SHP) and constitutive androstane receptor (CAR) are important components in BA signaling (22). The main receptors with their target and metabolic effects are shown in **Table 1**.

##### 3.1.1.1 Farnesoid X receptor

**Figure 2:**
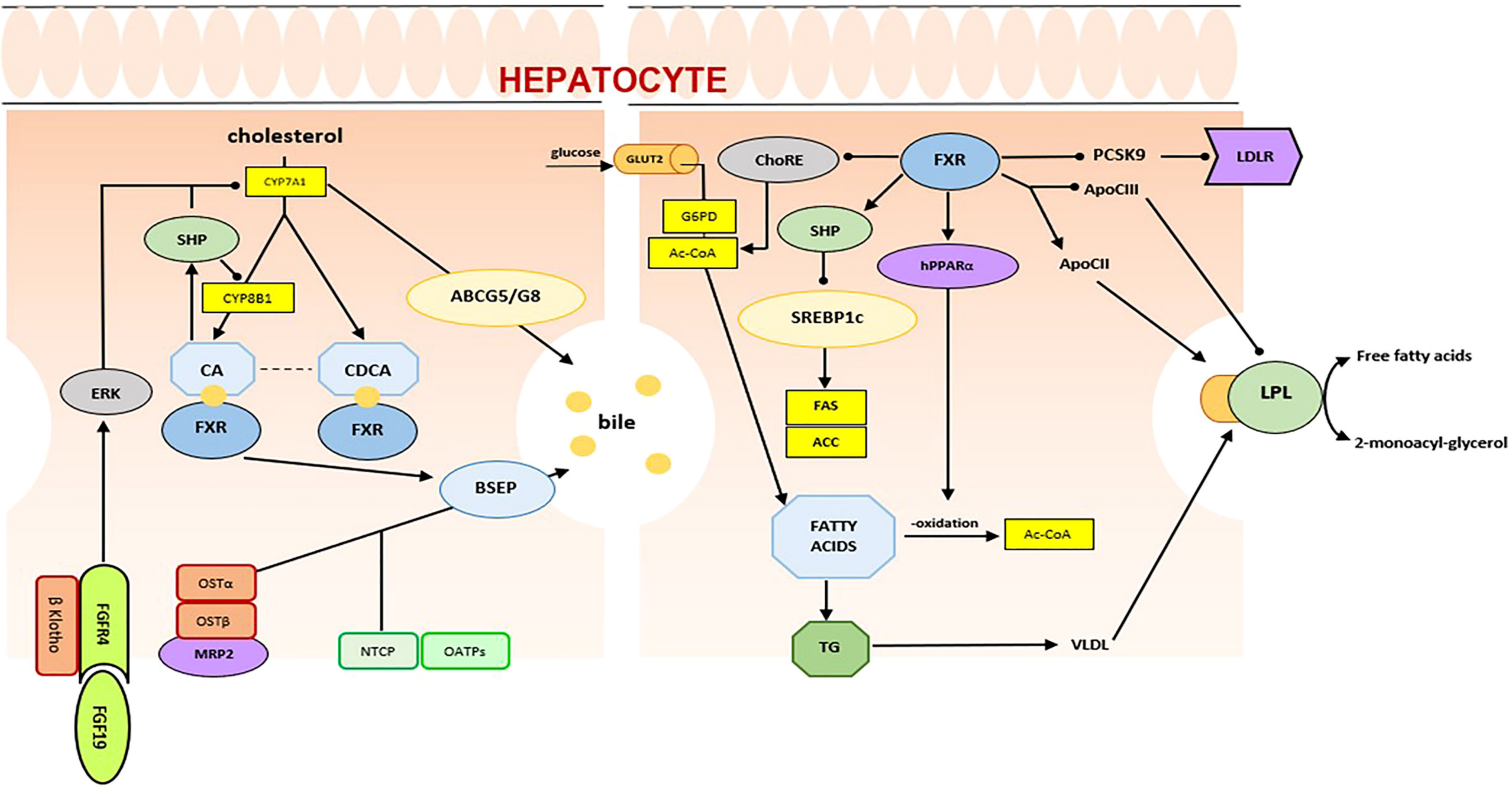
FXR in bile acid and lipid metabolism.

Firstly identified by Forman et al. in 1995 as a putative receptor for farnesol, an intermediate in cholesterol synthesis, the FXR is actually considered the primary BA sensor in humans (22, 23) (**Figure 2**). The FXR gene is mapped to chromosome 12q23.1 and produces four functional transcript variants, termed FXRα1 to FXRα4, that are generated by alternative promoter usage and alternative splicing (24, 25). Upon activation by its ligands, FXR interacts with its heterodimer partner Retinoid X Receptor (RXR) and binds to its specific DNA response element [FXR response element (FXRE)], regulating transcription of its target genes. As suggested by its high expression in the liver and intestine, FXR is a primary actor in the regulation of enterohepatic recycling of BAs and in the feedback regulation of BA biosynthesis.

FXRα inhibits BA synthesis in the liver through a feedback mechanism that involves SHP, an atypical NR that acts as a corepressor of CYP7A1, the rate-limiting enzyme in BA synthesis, and sterol 12-alpha-hydroxylase (CYP8B1), which is involved in BA synthesis. Moreover, in hepatocytes, FXRα controls intracellular concentrations of BAs by regulating its uptake and export. The final effect is the prevention of the accumulation of BAs and the protection of the liver from their potential toxic effects. Similarly, in the gastrointestinal tract, FXR reduces BA absorption to enterocytes and promotes their transport to the portal venous system. FXRα plays a significant role also in lipid metabolism, through regulation of hepatic lipogenesis, lipid oxidation, clearance, uptake, and transport (22). In detail, in the liver, FXRα has shown to inhibit some lipogenic genes, including sterol regulatory binding protein 1c (SREBP1C), stearoyl-CoA desaturase-1 (SCD-1), and acyl-CoA synthetase short chain family member 2 (ACSS2) (26). On the other hand, activation of FXRα stimulates fatty acid oxidation, through the expression of peroxisome proliferator-activated receptor α (Pparα) and of fibroblast growth factor 21 (FGF21) (27, 28). In addition, FXRα, through the coordinated regulation of ApoC2 and ApoC3, plays a crucial role in the improvement of lipid profile. It has been demonstrated that FXRα directly upregulates expression of ApoE, phospholipid transfer protein (Pltp), and very-low-density lipoprotein receptor (VLDLr) and induces expression of apolipoprotein C2 (ApoC2), reducing plasma triglyceride levels (29–31). Furthermore, several studies have shown that BAs and FXRα control glucose homeostasis. In the liver, BAs inhibit gluconeogenesis *via* the downregulation of phosphoenolpyruvate carboxykinase (Pck1) and glucose-6-phophatase (G6pc), and the activation of aldo-keto reductase 1B7 (Akr1b7) (32–34). As a consequence, BAs reduce plasma glucose levels. However, some aspects of these pathways are not yet fully understood. In the intestine, activation of the BAs/FXRα-related pathway induces fibroblast growth factor 19 (FGF19), a postprandial enterokine that increases hepatic glycogen synthesis (35, 36). Moreover, recent studies have highlighted that the intestine-restricted FXR agonist fexaramine improves insulin sensitivity, through the stimulation of TGR5-induced glucagon-like peptide-1 (GLP-1) (37).

##### 3.1.1.2 Pregnane X receptor

PXR is widely expressed in tissues with high metabolic activity, including intestine and liver, justifying its main function as a xenobiotic sensor (38). In 2001, LCA was identified as one of the ligand for PXR (39), and the ability to regulate genes involved in BA homeostasis has been highlighted. In fact, recent studies have shown that PXR, once activated by LCA, regulates the expression of CYP7A1, Oatp2, and CYP3A, protecting liver by toxicity of BAs (40). In hepatocytes, PXR promotes the conjugation of bilirubin by upregulating the expression of UDP-glucuronosyltransferase family 1 member A1 (UGT1) (41).

Regarding lipid metabolism, PXR seems to induce the accumulation of triglycerides in the liver through interaction with some lipogenic genes, such as SCD-1 and CD36; on the other hand, it inhibits fatty acid β-oxidation and ketogenesis through upregulation of carnitine palmitoyltransferase 1A (Cpt1a) and mitochondrial 3-hydroxy-3-methylglutarate-CoA synthase 2 (Hmgcs2) and repression of FoxA2 (42, 43). Finally, PXR directly inhibits CREB’s transcriptional activity, which induces G6pc, leading to a downregulation of hepatic gluconeogenesis (44).

##### 3.1.1.3 Vitamin D receptor

Recently, some studies have demonstrated that secondary BAs, such as LCA and its metabolite, can interact with VDR, suggesting its role in biliary homeostasis. VDR is predominantly expressed throughout the gastrointestinal tract including duodenum, jejunum, ileum, and colon. Here, upon activation by its ligand, VDR interacts with its obligatory partner RXR and induces expression of some genes, such as CYP3A and MRP3, involved in metabolism and transport of BAs (45, 46). Accordingly, VDR contributes to protect the intestinal barrier from BA toxicity. In hepatocyte, VDR interacts with hepatic nuclear factor 4α (HNF4α) and inhibits CYP7A1 gene transcription, limiting BA synthesis (47).

#### 3.1.2 GPCR signaling pathways modulated by BAs

The GPCR family includes over 800 receptors; however, currently only three GPCRs are known to interact with BAs: TGR5, muscarinic receptors, and formyl peptide receptors (FPRs) (48).

##### 3.1.2.1 TGR5

**Figure 3:**
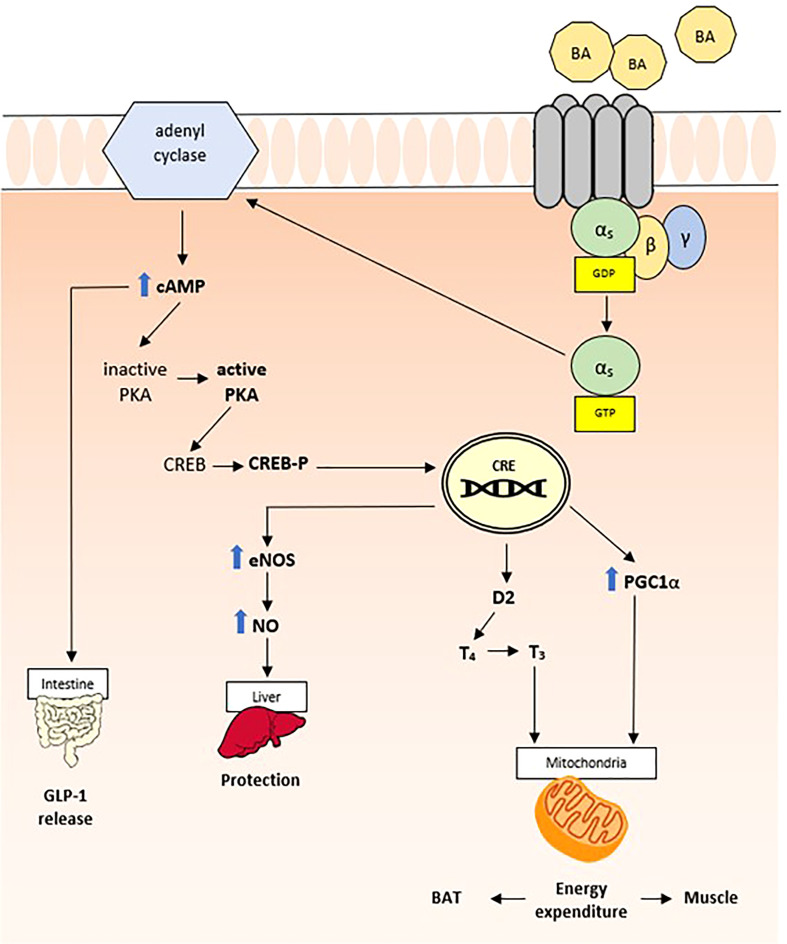
TGR5 related pathway.

In 2002, a novel category of BA receptor, TGR5, also known as M-BAR, GPBAR, or GPR131, has been identified (49). The TGR5 gene is mapped to chromosome 2q35 in humans, and similarly to other Gαs-type receptors, it promotes adenyl cyclase activity increasing c-AMP production and activation of MAPK pathways (**Figure 3**) (50). Despite the fact that it can be activated by multiple BAs, LCA is the most potent natural agonist (50). TGR5 expression levels vary between different tissues, with high expression levels in brown adipose tissue, liver, intestine, and selected areas of the central nervous system (51). Consequently, the biological impact of TGR5 activation by BAs is variable and, to date, has only been partially understood. Like other BA sensors, TGR5 contributes to the regulation of the BA pool and composition (37); however, multiple roles have been highlighted over the years. One of the most relevant properties of TGR5 is immunomodulation. In fact, it is highly expressed in monocytes and macrophages and in human spleen. Through TGR5, BAs increase cAMP and inhibit LPS-induced cytokine secretion, including tumor necrosis factor-α (TNF-α), interleukin (IL)-1α, IL-1β, IL-6, and IL-8 (50).

TGR5 is also widely expressed in Kupffer cells, the “hepatic” resident macrophages, which protect liver from steatosis, preventing non-alcoholic fatty liver disease (37). In addition, TGR5 protects liver against lipid peroxidation, regulating nitric oxide production *via* cAMP-dependent activation of endothelial nitric oxide synthase (eNOS) in sinusoidal endothelial cells (52). Finally, through GLP-1, TGR5 contributes to glucose homeostasis and insulin sensitivity. Although the mechanism behind TGR5-induced GLP-1 secretion is not yet fully understood, stimulation of oxidative phosphorylation could be the trigger for cell membrane depolarization and Ca^2+^ mobilization, resulting in the release of insulin from pancreatic β cells (53).

##### 3.1.2.2 Muscarinic receptors

Muscarinic receptors (M1–M5), expressed in the central nervous system and in peripheral organs, are engaged in numerous physiological processes, including smooth-muscle contractility, glandular secretion, and insulin release by pancreatic β-islet cells. Through them, BAs could contribute to a broad range of metabolic effects; however, further studies are needed to accurately establish the effects of this interaction (20).

##### 3.1.2.3 Formyl-peptide receptors

The FPRs are a group of G-protein-coupled receptors that play important roles in immune response and inflammation. In humans, three genes coding for FPRs have been cloned, including FPR1, FPR2, and FPR3. These genes are expressed on a broad spectrum of human tissue and cells, including neutrophils and monocytes (54). In 2000, Chen et al. have demonstrated that BAs can competitively inhibit the functions of FPR, suggesting that BAs contribute also to anti-inflammatory response (48).

## 4 BAs and obesity: Current knowledge

Fatty acids and glucose are two major constituents of the body. The ability of BAs to regulate a wide spectrum of metabolic pathways, including lipid and glucose metabolism, suggests the key role of these molecules in the modulation of energetic metabolism and in the pathogenesis of obesity. When meal arrives in the intestine, BAs reach the gut and induce synthesis of glucagon-like peptide 1, a powerful insulin-releasing protein. In patients with type 2 diabetes mellitus (T2D), a decreased gallbladder motility is directly related to reduced BA secretion into the gut and to reduced insulin release. High serum levels of insulin suppress BA *de novo* synthesis by inhibition of CYP7A1 expression, which is the rate-limiting enzyme in hepatic BA biosynthesis (55). Available data have shown that total plasma BA concentrations appear to be positively correlated with obesity, T2D, and NAFLD as evidenced by higher fasting or postprandial plasma BA levels.

In 2006, Watanabe et al. have shown for the first time that BAs increase energy expenditure in brown adipose tissue, preventing obesity and insulin resistance in mice (51). These effects seem to be critically dependent on TGR5, which, through the activation of the type 2 enzyme iodothyronine deiodinase (D2) in brown adipocytes and skeletal myocytes, leads to increased oxygen consumption (53). Subsequent studies have supported the importance of TGR5 as a key regulator of energy expenditure, thanks to the demonstration that TGR5 mice −/− show significant weight gain and fat accumulation (56) and that the administration of a TGR5 agonist to wild mice reduces obesity and glucose levels (57). However, in humans, the role of TGR5 in energy metabolism is still not clear. In fact, through *in vitro* studies, it has been demonstrated that TGR5 regulates energy metabolism even in human muscle cells, and,the levels of this receptor in human skeletal muscle, adipose tissue and intestine are very low (50). Together with TGR5, FXR also represents an essential receptor of energy metabolism. As discussed above, FXR-α directly regulates the expression of FGF19 in the intestine, which suppresses CYP7A1 expression into the liver in an SHP-independent manner (58). Transgenic mice overexpressing FGF19 show an improved metabolic rate and an attenuated weight gain, due to decreased ACC2 expression. Lower ACC2 expression reduces the level of malonyl-CoA that inhibits the activity of carnitine palmitoyl transferase 1, the rate-limiting enzyme controlling the import of fatty acids into the mitochondrial matrix, increasing liver β-oxidation and brown adipose tissue mass (20). Finally, it has been recently reported that FGF19 reduces insulin-induced fatty acid synthesis in hepatocytes by inhibiting lipogenic gene expression and PGC-1α (59). Shp null mice (*shp-/shp-*) show increased energy expenditure, PGC-1α expression, and diet-induced obesity, suggesting that SHP may be involved in energy production in brown adipose tissue by inhibiting PGC-1α expression (60).

### 4.1 Bile acid profile in obese young

Novel knowledge on the role of BAs in obese children and adolescents has been provided by a recent Italian study (61) that has observed the presence of impaired levels and composition of BAs in obese children with peculiar differences according to gender and the presence of T2D. In fact, in contrast to previous reports (62), the authors have shown a higher concentration of BAs, primary CA and primary glycine-conjugated (i.e., GCA and GCDCA) and taurine-conjugated (i.e., TCA, taurocholic acid) BAs, in male than in female patients, obtaining data similar to that collected from adult reports (63). The excess of adipose tissue, especially in obese children with hepatic steatosis, probably makes children more similar to adults and establishes an unfavorable metabolic profile predisposing to an atherogenic dyslipidemia characterized by qualitative changes in LDL and HDL cholesterol (61). It is established that this imbalance in lipid metabolism predisposes to a higher production of estrogens, whose synthesis is directly correlated to cholesterol levels, which can influence BA synthesis and pool composition. In fact, the interaction of estrogen with specific receptors localized on liver cells activates intracellular pathways that regulate the activity of some of the many enzymes involved in BA synthesis (64). Furthermore, estrogens mediate an inhibitory effect on BA transport within the liver, thus impairing the conjugation pathway and subsequently pool composition (63).

To support these data, glycine-conjugated BAs were also found to be correlated with AST (aspartate amino transferase) and ALT (alanine amino transferase), confirming a direct correlation between change in BA composition and liver involvement. NAFLD and obesity are two diseases highly correlated to each other, and concomitant to the increased rate of pediatric obesity worldwide, NAFLD has become the most frequent chronic liver disease affecting about 40%–80% of obese children (65). The pathogenesis of the disease is not yet fully understood, but a role of the components of the metabolic syndrome is recognized (66). In this context, BAs seem to be an important link between the metabolic syndrome, liver disease, and the gut through the enterohepatic circulation and the interactions between BA and insulin. The increased levels of primary and conjugated BAs in children with steatosis might derive from three major mechanisms, namely, increased synthesis of primary BAs, decreased conversion to secondary BAs in the intestine, and decreased bile excretion. It is known that, compared to CDCA, primary BAs and its conjugates have a lower activity as an FXR agonist (67), which is fundamental in glucose homeostasis (32). In fact, FXR-null mice display severe steatosis associated with increased serum glucose levels and impaired glucose and insulin tolerance (32). Therefore, the increased concentration of CA and its conjugates in children with NAFLD might promote insulin resistance, demonstrating the correlation between abnormal BA profile and the pathogenesis of liver disease. In addition, hepatic FXR-mediated and FGF receptor 4-mediated BA signaling is inhibited in obese patients (67). A pediatric animal model study performed by Hernandez et al. has demonstrated that a high-fat diet induces non-alcoholic steatohepatitis (NASH) in juvenile pigs and is associated with gut dysbiosis and with abnormalities of enterohepatic FXR–FGF19 signaling (68).

Therefore, BAs may be considered biomarkers of progression of hepatic alterations. The interest in BA derives from the evidence of significantly elevated serum BA levels in subjects with compensated cirrhosis (69). A pediatric Chinese study has suggested an increased serum concentration of CDCA and unconjugated BAs in the moderate and severe stage of NAFLD. In contrast, serum concentrations of DCA and conjugated DCA were downregulated in the mild stage. In addition, lower serum concentrations of GLCA and total LCA and higher n-7 MUFA (palmitoleic acid) have been reported in patients having both stages of NAFLD when compared with non-NAFLD subjects (70).

According to a study performed on European children, total BA levels in biopsy-proved NAFLD were confirmed to be lower than that observed in healthy controls. The authors postulated that these results are probably due to the fact that low glycine-conjugated BA levels were incompletely compensated by increases in taurine-conjugated or unconjugated BAs (66).

However, it is difficult to establish an independent association between BA alteration s and single disease. For example, Legry et al. (71) have demonstrated that BA alterations in obese patients with NASH were mainly related to insulin resistance rather than to the alterations of the liver. The importance of insulin resistance in BA metabolism has been demonstrated in different studies that showed the concomitant increase of insulin levels and a decrease of FGF19 levels in line with the development of fibrosis (66, 72). A study of Park et al. has suggested a relationship between insulin levels and BA synthesis. In fact, high levels of insulin reduce BA synthesis while increased levels of BAs reduce insulin release *via* GLP-1 (73). In addition, in patients with insulin resistance, the synthesis of intestinal FGF19 is reduced and correlates inversely with progression to liver fibrosis (66). Since serum BA levels are low in early NAFLD, the stimulus for FGF19 secretion is missing, leading to low serum FGF19 concentration. Although FGF19 suppresses BA synthesis, the main effect on the suppression of BA synthesis is exerted by insulin (66). Therefore, studies characterizing the link between obesity and BA are still missing and urgently needed in order to better understand this complex pathway.

### 4.2 Obesity, gut microbiota, and BAs

The human intestinal microbiota includes about 3,000–5,000 bacterial species that contribute to the regulation of several metabolic functions and energy balance, with long-lasting health consequences. Although changes in the gastrointestinal microbiota have been described in association with obesity in mice and humans, the exact mechanisms by which microbial functions influence the host energy metabolism and adiposity are not completely described (74–78).

A role of gut microbiota as a microbial metabolic organ has been proposed due to its ability to regulate the energy balance derived from ingested foods and to promote the release of gut hormones. However, much more should be known about the relationship between microbiota alterations and metabolic disorders.

The composition of gut microbiota, acquired at birth from the mother, remains relatively stable during childhood, with a prevalence of Bacteroides and Firmicutes. However, different factors such as mode of delivery, diet, and breastfeeding can cause changes in the gut microbiota, which, in turn, can increase the risk of pediatric overweight or obesity and long-term health consequences (79). A significant variation in quality has been evidenced in some pathological conditions, such as obesity and metabolic syndrome. The observation that the gut microbiome presented a lower richness of bacterial communities in obese than lean individuals (75) and the evidence that a reduction in bacterial composition has been associated with a marked overall adiposity, insulin resistance, and dyslipidemia compared with individuals with high bacterial richness (80) have offered the basis to evaluate the real link existing between microbiota and obesity.

Studying the microbiota profiles of obese children and adults, it has been shown that they differ from each other supposing a combined and independent role of age and obesity in determining the microbiota population. In particular, the pattern of microbiota in obese adolescents is enriched by lipopolysaccharide metabolism, which promotes the inflammation-related processes associated with the onset of obesity and insulin resistance. In contrast, the microbiome in obese adults compared with normal-weight controls is involved in the pathways of the initial steps in breaking down indigestible dietary polysaccharides (76, 81).

Analyzing microbiota composition, *Firmicutes*, *Bacteroidetes*, *Proteobacteria*, and *Actinobacteria* are the most predominant bacterial phyla in the human gut microbiota (82, 83), with a prevalence of the first two phyla. The former are Gram-positive bacteria to which *Clostridium*, *Lactobacillus*, and *Coprococcus* belong. Instead, *Bacteroidetes* includes *Bacteroides*, *Prevotella*, and *Desulfuribacillus*. Greater differences arise by comparing microbiota composition of obese and normal weight in both adult and pediatric population. In particular, *Firmicutes* increases in overweight/obese to the detriment of *Bacteroidetes*, which decreases (76, 84), resulting in an increase in *Firmicutes*: *Bacteroidetes* ratio (85). In contrast to normal-weight children and adolescents, a positive correlation exists between *Firmicutes* levels and BMI (86, 87) in overweight/obese subjects. Differently, *Bacteroidetes* correlates negatively with BMI (88, 89).

Although the *Firmicutes : Bacteroidetes* ratio has been positively associated with pediatric overweight/obesity (90, 91), its use in clinical practice as an index of gut microbiota composition and as therapeutic target is now debated. In particular, several studies have questioned the possibility that the abundance of *Firmicutes* and *Bacteroidetes* was subjected to a great individual variation, exacerbated in the pediatric age by a different bacteria composition during physiological development. More practical information might be obtained by studying gut microbiota at the genus level or evaluating specific metabolites (90). Regarding the epigenetic effects of *Firmicutes*, the prevalence of bacteria belonging to this phylum has been associated with DNA methylation of genes related to lipid metabolism, inflammatory response, and obesity (92). Moreover, the abundance of *Firmicutes* was positively associated with an inflammatory state and to higher serum tumor necrosis factor α (TNF-α) levels in obese children (87). Therefore, those markers might be used as an alternative tool of gut microbiome composition.

*Proteobacteria* represents a phylum of gut microbiota to which species able to initiate the inflammatory burden belong (93). Those bacteria are highly abundant in the feces of obese children with a positive correlation with BMI levels (94, 95). However, *Proteobacteria* are also relatively abundant among malnourished children, with decreasing levels inversely to physical exercise (96). Among them, *Gammaproteobacteria*, especially *Enterobacteriaceae* to which *Escherichia coli* belong, participate in the metabolism of choline and they are particularly expressed in overweight/obese children with NAFLD (97–99). In accordance, the abundance of *Escherichia coli* is also significantly higher in obese children (100).

The richness of *Bifidobacterium* is negatively correlated with BMI in children. A higher amount of *Bifidobacterium* has been documented in overweight/obese children than in children of normal weight. It has been hypothesized that they participate in fat accumulation and obesity (86). As evidence of this, during weight loss, *Bifidobacterium* abundance rebounds (101). Moreover, *Bifidobacterium infantis* metabolizes human milk oligosaccharides (HMO) and reduces HMO uptake by pathogenic microbes (102).

Bacteria belonging to *Verrucomicrobia* phyla have been described as bacteria potentially influencing human metabolism. This phylum is relatively rare in the human gut microbiota. However, recently, a reduction in the abundance of *Verrucomicrobia* has been observed in obese children (103–105). The anaerobic bacterium *Akkermansia muciniphila* is the only known member of *Verrucomicrobia* in human’s intestinal tract (106). As well as adults (107), overweight/obese children have lower *A. muciniphila* abundance (108), suggesting a non-casual association. Recently, *A. muciniphila* has been described as an important species in the intestinal environment in which abundance seems to reduce diet-induced obesity (106). However, its clinical implication in the pathogenesis of obesity needs to be validated by further studies.

Finally, greater differences arise by comparing microbiota composition of obese and normal-weight adolescents. In particular, following the operational taxonomic unit (OUT)-based model, *F. prausnitzii* and *Actinomyces* are more represented in obese adolescents, while *Parabacteroides*, *Rikenellaceae*, *B. caccae, Barnesiellaceae*, and *Oscillospira* characterize the normal-weight control (81). According to another study, the reduced levels of *Bacteroides thetaiotaomicron*, which is important for glutamate metabolism, results in a higher risk of obesity (109).

These preliminary data about the link between microbiota and obesity have increased the focus from the scientific community to mainly identify the possible factors responsible for those differences. Recently, the diversity in microbiota population between infant with cholestasis has opened the possibility of identifying a link between microbiota and BAs. An important reduction of *Bacterioides* and *Firmicutes* and a marked increase in the proportion of *Proteobacteria* have been documented in children with obstruction in the efflux of bile (110). However, different richness has been identified within children with different types of cholestasis. In particular, a relative abundance of *Proteobacteria* in biliary atresia (BA) and lower richness of *Bacterioides* and *Firmicutes* compared to the non-biliary atretic cholestasis group (CD) have been observed, suggesting more severe gut microbial dysbiosis in the first group. By analyzing gut microbiota before and after surgery, with the excretion of BA s, potential probiotics were found to increase significantly than preoperatively, offering the possibility to establish a precocious and faster recovery of intestinal microbiome population with supplementation of potential probiotics (110).

Although different defense mechanisms have been highlighted in intestinal microorganisms (i.e., active efflux system, DNA repair, and cell envelope remodeling), it is known that BAs can directly and indirectly influence gut microbiota composition through bacteriostatic and bactericidal effects and regulation of host immunity (67, 111–113).

BAs themselves have antimicrobial activity by damaging the bacterial cell membrane and by suppressing bacterial overgrowth under normal conditions. In a high-fat diet, *Bacteroides*, *Alistipes*, and *Bilophila* decrease, while polysaccharide digesting bacteria, such as *Roseburia*, *Eubacterium rectale, Ruminococcus bromii*, and *Firmicutes*, increase (114). Nevertheless, despite this evidence, the real connection between diet and gut microbiota remains unclear. Studies evaluating the effects of probiotics and prebiotics have shown a reduced permanence of bacterial administered in the gut not allowing to obtain useful results (115).

Conversely, intestinal microorganisms can modify the composition of BA pool through various reactions, including deconjugation, epimerization, and dihydroxylation or converting primary BA into secondary BA (116). In particular, the 7α-dehydroxylation reaction, which has a key role in the biotransformation process of BAs into secondary BAs, has been described as the most quantitatively important process performed by gut microflora (17). Although over the years the role of other bacterial genera has been clarified (117), some studies have shown that only bacteria belonging to the genus *Clostridium* can catalyze the 7α-dehydroxylation reaction, thanks to the BA -inducible operon (BAI), which is highly conserved in both *Clostridium scindens* and *Clostridium hylemonae* strains (17, 118). Consequently, changes in the composition of the BA pool determine changes in the microbiota and *vice versa*.

During the last few years, the involvement of the FXRα has been assumed. In fact, recent work has demonstrated the significant activation of the FXR-related pathway in mice inoculated with *E. coli* that overexpressed the active allele of bacterial bile salt hydrolase (BSH), mediating a microbe–host dialogue that functionally regulates lipid metabolism in the host. In gnotobiotic or conventionally raised mice, BSH enzymes in the gastrointestinal tract significantly modify plasma BA signatures and regulated transcription of key genes involved in lipid metabolism (*Pparγ* and *Angptl4*), cholesterol metabolism (*Abcg5/8*), gastrointestinal homeostasis (*RegIIIγ*), and circadian rhythm (*Dbp* and *Per1/2*) in the liver or small intestine. Therefore, the increased expression of BSH in conventionally raised mice brings significant reduction in weight gain, plasma cholesterol, and liver triglycerides. Further studies might confirm the pathogenetic role of BSH, offering a future potential therapeutic target for the control of obesity and hypercholesterolemia (119).

In conclusion, the alteration in the composition of the BA pool can be associated with a different gut microbiota composition in obese young. At the same time, intestinal bacteria belonging to different phyla could themselves influence the BA composition in a reciprocal relationship. Therefore, it is deemed that both simple and genetic obesity [such as Prader–Willi syndrome (PWS)] can benefit from interventions that aim to improve the imbalance of the gut microbiota to intervene against overweight/obesity in children.

## 5 Therapeutic strategies

The large correlation between BAs and metabolic disorders offers the opportunity to expand the treatment options for them. In this direction, the use of BAs, BA -binding resins, and synthetic FXR and TGR5 agonists could interfere with the cascade of events responsible for the elucidation of the clinical picture (120). However, their real functionality and applicability in humans are discussed since they might have variable effects on the overall BA pool size and composition (121). The type and/or the severity of the underlying disorder probably influences the starting BA pool with different responses to the treatment and the possibility to determine adverse effects (5). Additionally, FXR and TGR5 are expressed in many body tissues regulating a lot of pathways (5). Therefore, it is necessary to identify molecules that are able to act selectively on target tissues to reduce the possibility of developing adverse effects. Finally, most of the current knowledge derives from mouse models that could have a different BA composition than humans (122). Specifically, taurine conjugates two major BAs in mice [a- and b-muricholic acids (Ta/bMCA)], studied as FXR antagonists, which, however, are not present in humans (123, 124). In contrast, in humans, unconjugated and taurine-conjugated lithocholic acid (LCA/TLCA) act as agonists of TGR5, but they have not been found in mice. Therefore, all results obtained in mice cannot be applied to humans (125). In the following paragraphs, we will discuss the potential therapeutic target. Unfortunately, all reported molecules are not yet approved for the treatment of obesity in both adults and children/adolescents with obesity.

### 5.1 Bile acid binding sequestrants

The use of BA binding sequestrants, including cholestyramine, is founded on the evidence that they induce a decrease in circulating lipids by suppressing the enterohepatic circulation of BAs in the ileum. Therefore, a large amount of primary BAs go to the large bowel where they are changed into secondary BAs, mostly lost from the digestive tract. To compensate for the intestinal loss, BA synthesis is activated in the liver from cholesterol with consequent reduction of serum LDL cholesterol concentrations (126). However, it has been described that long-term BA binding sequestrant treatment leads to hypertriglyceridemia (126). The reason could rely on gut dysbiosis secondary to increased BA levels in the intestinal lumen. In fact, BAs in the gut are metabolized into secondary BAs by gut microbiota, which, in turn, modulates several metabolic pathways to influence host metabolism (127).

### 5.2 Drugs that target bile acid receptors

#### 5.2.1 FXR agonists

BAs, interacting with FXR in the liver, suppress hepatic lipid biosynthesis and glucose metabolism. These characteristics make FXR a druggable target for the treatment of obesity and cardiovascular diseases. BAs are considered important endogenous FXR agonists in different tissues. The potency of natural BAs to activate FXR follows the order CDCA > DCA > LCA > CA (128). The first FXR agonist that was investigated in clinical practice was obeticholic acid (OCA), which has an ethyl group substituted at the 6 position of CDCA, which is the most potent natural ligand activator of FXR (129). OCA was approved by the United States (US) Food and Drug Administration for the treatment of primary biliary cholangitis (PBC) and evaluated in clinical trials for non-alcoholic steatohepatitis (NCT01265498), alcoholic hepatitis (NCT02039219), and lipodystrophy (NCT02430077). Currently, its use is limited by the appearance of pruritus.

INT-767 is a semisynthetic BA that activates both FXR and TGR5. It has been described as the most powerful activator of FXR, which also has the ability to alleviate liver disease and metabolic disorders. INT-767 has been shown to alleviate hypercholesterolemia and increase the expression of thermogenic genes through FXR and/or TGR5 activation, leading to the reversal of HFD-induced metabolic disorders (130).

TC-100 (3α,7α,11β-trihydroxy-6α-ethyl-5β-cholan-24-oic acid) is the first semisynthetic BA that combines the ability to specifically bind and activate FXR without TGR5 activation (131). The study of the activation of FXR by TC-100 through cell-based analysis revealed that TC-100 is slightly more potent than OCA and is highly effective in increasing the clearance of BAs from the liver to the bile canaliculus (131).

Among all the agonists studied in this field, there is also FGF19. It has been proposed as a candidate to treat NASH and obesity-related disorders, to the detriment of increasing the risk of cancer. In this regard, the recombinant FGF19 mimetic peptide NGM282 that has been tested *in vivo* to treat patients with metabolic liver disease does not increase the risk of cancer. Surprisingly, anti-sense FGFR4138 (ISIS) has also been described as potentially effective to induce fat burning and energy expenditure in mice. However, the differences between mice and humans do not allow the translation of these results in clinical practice. Therefore, further studies are needed to expand this field of research to medicine.

#### 5.2.2 TGR5 agonists

TGR5 (also known as Gpbar-1), a G- protein-coupled BA receptor, is a potential drug target useful to treat obesity and associated metabolic disorders. Its activation results in energy expenditure in the brown adipose tissue (BAT), an increased GLP-1 secretion by enteroendocrine L cells, and simultaneously anti-inflammatory activity. TGR5 agonists have been proposed as a good therapeutic strategy to treat patients with obesity and T2D. In this regard, two semi-synthetic BA derivatives have been detected: INT-767 (53) and non-steroidal TGR5 agonists (132, 133). However, the improving glucose homeostasis has to deal with the appearance of two adverse effects: an increase in gallbladder volume (132, 133) and pruritus (134). Therefore, efforts should begin to focus in researching molecules that selectively act as a topical intestinal TGR5 agonist with the result of preserving GLP-1 secretion and avoiding cholecystomegaly but, at the same time, risking to lose the effect on BAT.

Recently, a study conducted by Ding and colleagues in mice has identified notoginsenoside Ft1 (Ft1) fas, an agonist of TGR5 *in vitro* (135). It has been revealed that the administration of 100 mg of FT1 in diet-induced obese mice leads to adipose lipolysis and promotes fat browning in inguinal adipose tissue and glucagon-like peptide-1 (GLP-1)-increased secretion. Furthermore, Ft1 acts as an antagonist of *FXR* transcriptional activities in the ileum to activate *TGR5* in the adipose tissues, and as a result, it elevates serum-free and taurine-conjugated BAs. However, all the above mentioned metabolic effects are not evident in *Cyp27a1−/−* mice, which have lower BA levels. Considering the double action of Ft1 on TGR5 and FXR, it might be used to treat obese subjects with insulin resistance (135).

#### 5.2.3 GLP-1 receptor agonists

Intestinal lipid homeostasis is dominated by a complex neuroendocrine network involving gut peptides, namely, glucagon-like peptide 1 (GLP-1) and 2 (GLP-2). GLPs are cosecreted from enteroendocrine L cells after nutrient intake (55). It is known that GLP-1 leads to weight loss by delaying gastric emptying, promoting satiety, and reducing food intake (136).

GLP-1 Ras was originally designed and distributed for its effect on glycemic control, with reductions in HbA1c. In adults, meta-analyses of randomized controlled trials (RCTs) of GLP-1 Ras in patients with T2DM and obesity have shown benefits on glycemic control and weight loss. Currently, GLP-1 Ras is approved for use in T2DM in adults and in children aged higher than 10 years by the US FDA (137) and for weight management in adults by the FDA and European Medicines Agency (EMA) (138). GLP-1 Ras therapy seems to be well tolerated in children, with only mild side effects. The most common adverse effects involve gastrointestinal system, manly nausea, as aconsequence of the effects of GLP-1 on delaying gastric emptying (139).

In term of effectiveness, although it has been demonstrated a significative weight loss effect in pediatric patients treated with GLP-1 Ras, there was no difference in the efficacy of liraglutide and exenatide in obese children and adolescents (140).

A randomized, double-blind, placebo-controlled trial was performed on 21 subjects, aged 12–17 years and Tanner stage 2–5, with obesity. It has demonstrated the same safety and tolerability profile of liraglutide in both children and adults with obesity, with no unexpected safety/tolerability issues (141). Results of this study suggest that the dosage approved for weight management in adults may be appropriate for use in adolescents. Another study by Kelly et al. has explored the effects of GLP-1 Ras on body mass in obese adolescents (142). First, the authors have highlighted the mean percent change in BMI measured at baseline and 3 months. Second, they demonstrated absolute change in BMI, body weight, body fat, blood pressure, hemoglobin A1c, fasting glucose, fasting insulin, and lipids after 3 months (142).

In conclusion, despite being promising, these studies need to be implemented by larger trials with longer periods of treatment in order to evaluate, in the long term, the weight loss effect over time of these medications.

### 5.3 BAs and bariatric surgery

In severely obese patients with comorbidities, one effective current available therapeutic approach is bariatric surgery. It primarily targeted weight loss by inducing, malabsorption of, or restricting the gastrointestinal tract, depending on the technique adopted. Over the years, it has been shown that bariatric surgery leads to not only weight loss, but also an improvement in metabolic profiles, especially in insulin sensitivity, from the first days after surgery (143), so as to be renamed “metabolic and bariatric surgery” (MBS) (144). Because this effect cannot be attributed to weight loss that appears lately, it has been supposed that other factors might mediate this effect, and among them, interestingly, BAs have emerged as one of the most important mediators (145).

This surgical strategy is a standard treatment for obese adult patients, but recently, it has also emerged as an option for adolescent patients. However, the lack of definite and unified guidelines restricts the use of this interventional strategy for the entire population of obese children and adolescents. The American Society for Metabolic and Bariatric Surgery (ASMBS) Pediatric Committee in the USA recommends bariatric surgery for class II obese subjects with comorbidity (such as non-alcoholic liver steatosis, T2DM, obstructive sleep apnea syndrome, cardiovascular risk factors, orthopedic disease, physical impairment, gastro-esophageal reflux disease, idiopathic intracranial hypertension, and low quality of life) or class III obese subjects without comorbidity (146). In 2014, the National Institute for Health and Care Excellence (NICE) in the UK proposed bariatric surgery for patients with a BMI of 40 without comorbidities, for patients with a BMI of 35 with comorbidities, or for patients with a BMI of 30 who have new-onset T2DM (147). In 2015, the European Society for Paediatric Gastroenterology, Hepatology, and Nutrition (ESPGHAN) suggested the use of bariatric surgery to adolescents with a BMI ≥40 kg/m^2^ in association with severe comorbidities or those with a BMI ≥50 kg/m^2^ with mild comorbidities (148). Unfortunately, the MBS use for adolescents is limited due to the possible appearance of side effects on growth and puberty beyond the risk of nutritional deficiencies and osteoporosis related to the malabsorption induced by the surgical intervention (149, 150). However, several recent high-quality studies of adolescents treated with MBS have reported encouraging data regarding the effects of MBS in both weight loss and cardiometabolic improvement, with a safety profile similar to that seen in adult patients (151–155). The actual NICE and the ESPGHAN guidelines approve the use of bariatric surgery only for patients who have achieved physical maturity or adult height (147, 148). Conversely, ASMBS guidelines propose an integrated eligibility approach where the Tanner pubertal stage and linear growth do not exclude patients from bariatric surgery (146).

The limitations in the use of this therapeutic strategy does not allow obtaining data regarding the existing relationship between bariatric surgery and BA changes in children and adolescents. Nonetheless, the knowledge of metabolic changes in adults might offer an opportunity to extend the eligibility criteria for surgical treatment to an additional part of the pediatric population.

Several studies report different BA profiles attributed to the different techniques used. The most popular surgical procedures are Roux-en-Y gastric by-pass (RYGB) and vertical sleeve gastrectomy (VSG); less common procedures include biliopancreatic diversion and laparoscopic adjustable gastric banding (LAGB). In adult patients treated with RYGB bariatric surgery, circulating BAs are increased under both fasting and postprandial conditions simultaneously to an elevation of 12a-hydroxylated/non-12a-hydroxylated BA ratios. Similar changes have been documented after biliopancreatric diversion with duodenal switch (145, 156). Conversely, VSG has no clear effects on BA pool while LAGB does not seem to affect its quality and consistency. Not surprisingly, these last two techniques are not associated with an improvement in glucose metabolism compared to RYGB (157). Currently, scientific justifications about these differences are not yet available. In patients who underwent RYGB and VSG, a parallel elevation of FGF19, step-down regulator of BA synthesis, and BA levels has been observed. Otherwise, BA synthesis, as estimated by 7a-hydroxy-4-cholesten-3-one (C4) level, is decreased in VSG, biliopancreatic diversion, and RYGB (158, 159). The reason why there is a discrepancy between FGF19 levels and circulating BAs is of scientific interest. It has been supposed that gut microbiota might play a role, which could modify the primary and secondary circulating BA ratio as well as unconjugated and glycine- or taurine-conjugated BA ratio in the gut. As a consequence, due to a different receptor affinity to SLC10A1 (NTCP) and SLC10A2 (apical sodium-dependent bile salt transporter; ASBT), in the liver and in the gut, respectively, there is an altered re-uptake rate (160). Another hypothesis is that BAs interact with other metabolically active peptides including adiponectin, peptide YY, and GLP-1, which indirectly promote the activation of TGR5 through secondary BAs (161). However, to date, there are no studies that have documented a correlation between gut microbiota and TGR5-activated metabolites in subjects who underwent bariatric surgery. That does not mean that microbiota does not play a role in the BA-induced metabolic change after surgery for obesity (5). Several studies have documented changes in the gut microbiota composition as early as 3 months after bariatric surgery (162, 163), and in particular, these changes persist 9 years after surgery (162). These changes in bacterial intestinal population not only affect postprandial BA levels but also are responsible for reduced fat gain in transplanted GF mice, revealing a direct role of gut microbiota in the metabolic effect of bariatric surgery (162).

Ryan et al. (164) have shown that FXR is one molecular target of bariatric surgery. In fact, studies conducted on mice lacking FXR have documented a reduced body weight loss and less improvement in glucose tolerance than wild-type mice after VSG. Thus, these effects suggest that FXR has an important role in the metabolic improvement after bariatric surgery (164). Results in mice have shown reduced FXR signaling after RYGB surgery (165).

Such differences may be due to the different surgical techniques between VSG and RYGB (predominantly malabsorptive vs restrictive). VSG surgical procedure retains the physiological secretion of bile in the duodenum at the papilla of Vater, while BA s after RYGB are secreted in the jejunum through a surgically created anastomosis with the possibility of establishing a different intestinal environment for resident bacteria. Therefore, these anatomical differences need to be considered to evaluate the diverse effects on the metabolic features in humans (5).

Further animal studies have been conducted to evaluate a possible connection between TGR5 and bariatric surgery. Mice knockouts for TGR5 who underwent VSG have a reduced metabolic improvement despite a conserved weight loss (166, 167). Although there are no data to explain this phenomenon, this evidence should be used as a starting point to evaluate the influence that metabolic disorders, bariatric surgery, and genetic background have on each other. Further studies evaluating the role of bariatric surgery in achieving better metabolic health are necessary. Of support could be a better knowledge of FXR/TGR in specific tissue to increase the possibility to know the pathways activated in different surgical interventions (168).

### 5.4 Probiotics and prebiotics

The latest discoveries about the possible role of microbiota in the pathogenesis of several diseases have offered the opportunity to use probiotics and prebiotics as a prevention and/or therapeutic strategy. The term “ probiotics” refers to “live microorganisms that, when administered in adequate amounts, confer a health benefit on the host”, whereas prebiotic includes “substrate that is selectively utilized by host microorganisms conferring a health benefit” (169).

The use of probiotics, as mentioned in previous paragraphs (see the *Obesity, gut microbiota, and BAs* section), aims to invert the *Firmicutes: Bacterioides* ratio, to increase the abundance of *Actinobateria*, and to reduce the amount of *Propionobacterium*. Among the probiotics actually available, *Bifidobacterium* and *Lactobacillus* are certainly the best studied.

*Bifidobacterium* is a well-known probiotic of *Actinobacteria*, which is able to promote the development and maturation of intestinal mucosa during the first years of life, reducing the incidence of diarrhea (170). Furthermore, bacteria belonging to this phylum offer a protection against adverse microbiota through a competitive colonization, particularly contrasting the growth and proliferation of Enterobacteria and Enterococci (171). For these properties, an integration of *Bifidobacterium*, as a dietary supplement, has been attested as a possible strategy against pediatric obesity (172). Additionally, an integration of *Bifidobacterium breve BR03* and *B632* has been associated with a significant improvement in insulin sensitivity in obese children and adolescents (173), while a reduced inflammatory response has been observed in obese children with insulin resistance (IR) treated with *Bifidobacterium pseudocatenulatum CECT 7765* (174). Therefore, those probiotics could offer a double protection against obesity and diabetes.

*Lactobacillus*, a bacterium belonging to the phylum *Firmicutes*, is largely used in clinical practice as a probiotic (175). However, its usefulness in treating patients with pediatric overweight/obesity is discussed since the abundance of *Lactobacillus* has been described to be associated with an increased risk of pediatric overweight/obesity (176, 177) and concurrently a positive association between fecal *Lactobacillus* concentrations and serum C-reactive protein in children (91). However, certain members of *Lactobacillus*, such as *Lactobacillus paracasei*, have been described as protective factors against obesity in children with an unhealthy diet and therefore could be taken into account as a therapeutic strategy for obese children and adolescents (101, 178). Interestingly, studies carried out in high-sucrose diet-induced obese rodents have reported a favorable effect of *Lactobacillus gasseri* spp. in suppressing body-weight and fat-mass gain and in reducing fasting glycemia in *db*/*db* mice (179). Other probiotics, such as *Lactobacillus casei*, can also increase the abundance of *Bifidobacterium* in obese children, offering a synergistic effect (180).

Although the role of *Verrucomicrobia* phyla is yet to be clarified in the pathogenesis of obesity in children and adolescents, the recent evidence about the inverse relationship between their abundance and the prevalence of obesity in children could be an option to treat patients with an unhealthy metabolic profile (181).

Finally, a study performed with a commercial combination of probiotics (VSL#3: *Lactobacillus* spp., *Bifidobacterium* spp., and *Streptococcus thermophilus*) has documented that VSL#3 assumption reduces the hepatic inflammation caused by HF diet in young mice (182).

Although administration of live microorganisms (probiotics) seems to reduce obesity and related metabolic disorders, the exact mechanisms implicated in the beneficial effects of probiotics are not completely understood. It has been suggested that probiotics, in part, might reduce the pathways correlated to obesity such as the deposition of fat in adipose tissue and liver and the resulting secondary inflammation. One trigger for metabolic disease relates to the gut microbiota’s role in modulating inflammation whereby elevated circulating lipopolysaccharide (LPS), which is exacerbated by a high-fat or high-fructose diet, induces a low-grade inflammatory state termed metabolic endotoxemia (183). A change in metabolite production is also observed with dysbiosis and particularly for fecal BA s (FBAs), which require the gut microbiota for transformation (184). There is increasing interest in evaluating whether the modulation of the gut microbiota can improve obesity and metabolic homeostasis. Consumption of prebiotics is one such strategy. However, it seems that prebiotics play an important role. The most studied are highly fermentable carbohydrates that contrast several metabolic pathways involved in obesity and metabolic syndrome including hyperglycemia, inflammation, and hepatic steatosis, at least in animal models. It is not yet known how they mediate those effects. The most recognized hypothesis is that they may influence microbiota in both composition and function. However, to better appreciate those effects, human intervention studies with “colonic” nutrients (dietary fibers, prebiotics, and others), which allow the selection of beneficial bacteria, or with food containing colonic nutrients are necessary to evaluate the real importance of those nutrients in the nutritional management of overweight and obesity, in both adults and children (115).

A recent study by Nicolucci et al. has evaluated the role of prebiotics in treating obese/overweight children. Their study was a double-blind, placebo-controlled trial conducted on children, 7–12 years old, with overweight or obesity (>85th percentile of body mass index) but otherwise healthy. Twenty-two children were assigned to the oligofructose-enriched inulin (OI) group, while 20 children were assigned to the placebo group that has been offered maltodextrin. After 16 weeks of treatment, the authors have observed normalization of weight, reduction of body fat, modifications of primary fecal BAs, and selective variations of gut microbiota with a significant increase in *Bifidobacterium* spp., particularly an increase in the species of genus *Bifidobacterium* and a decrease in *Bacteroides vulgatus* in the OI group when compared with controls. No difference in the level of fecal primary BAs has been observed in participants of the OI group compared to the placebo group (185).

The increase in *Bifidobacterium* and *Lactobacillus* was associated with prebiotics administration and in turn with beneficial effects on metabolism. A lot of studies on the adult population have been performed. In a systematic review of clinical trials, prebiotic intake was associated with a significant improvement in satiety, postprandial glucose, and insulin concentrations in adult subjects (186). However, these promising outcomes in adults justify the assessment of prebiotics as a dietary intervention to modulate gut microbiota and metabolic outcomes also in obese children. In fact, the potential role of prebiotics that influences body weight in children was suggested by the slow rate of weight gain observed in a trial assessing combined prebiotic and calcium intake in non-obese healthy children performed by Abrams and colleagues. The authors have postulated a reduction in total fat mass in normal-weight and overweight children consuming 8 g of OI with supplemental calcium for a year (187). To date, however, there is no research assessing the totality of changes in gut microbiota in children with overweight and obesity with prebiotic intervention.

Regarding the relationship with BA s, increased serum levels of the primary BA were seen in obese patients with T2D. Different studies have documented a positive correlation between CDCA and BMI, HbA1c, LDL-cholesterol, and triglycerides (188). The study performed by Nicolucci et al. has also demonstrated no change in fecal CDCA percentage in the OI group, but there was a significant 17% increase in the placebo group over time. This mechanism probably derives from the fact that the intake of OI diminished the natural increase of primary FBAs seen in the placebo group as a consequence of increased *Bifidobacterium* (185).

Prebiotics are an inexpensive and non-invasive treatment; therefore, they may be a good alternative treatment for overweight and obese patients. The metabolic and microbial improvement demonstrated by few studies needs to be implemented in a larger clinical trial in volving a pediatric population.

## 6 Conclusion

Obesity and its related disorders (cardiovascular disease, diabetes mellitus, NAFLD, and cancer), in parallel with socio-economic and cultural changes involving both developed and developing countries, have become one of the most important issues involving children, adolescents, and adults worldwide. Nowadays, the scientific interest aims to explore other factors implicated in the pathogenesis of those diseases to identify new therapeutic targets. Given the recent knowledge on the possible role of BAs as regulators of metabolism, the pathways related to them should be considered to obtain a successful and personalized therapy for obesity and its related diseases. BAs promote a reduction in cholesterol levels and in liver fat accumulation, which parallel s the decreased inflammation rate and ER stress. Although BA levels have been positively associated with an improvement in metabolic state, there is no clear BA profile or component that is specifically associated with the disease. This could be attributed to a wide range of circulating BA levels depending on physiological and pathophysiological variables. However, most lines of evidence support the idea that both fasting and postprandial circulating levels increase in metabolic diseases, maybe as a compensatory mechanism. Moreover, there is no clear explanation yet regarding the complete mechanism by which BAs mediate their beneficial effects, and this hampers the possibility of finding an imminent advantage in the therapeutical field. Although many new drugs have been synthesized and tested, none of them could be applied in practical terms because there is no molecule that could selectively act on target tissues, resulting in varying effects on pool size and composition of BAs. Moreover, the activity of the major BA receptor FXR appears to differ in different disease states, affecting the response to BA manipulation.

In conclusion, the complex and complete role of BAs in human metabolism needs to be accurately evaluated in more depth through randomized controlled clinical trials with specific BAs and derivatives able to interfere with the cascade of events leading to metabolic dysregulation characterizing obesity and its related disorders.

## Author contributions

CM, SS, and CGe wrote the draft. CGi and FC revised the text. All authors contributed to the article and approved the submitted version.

## Conflict of interest

The authors declare that the research was conducted in the absence of any commercial or financial relationships that could be construed as a potential conflict of interest.

## Publisher’s note

All claims expressed in this article are solely those of the authors and do not necessarily represent those of their affiliated organizations, or those of the publisher, the editors and the reviewers. Any product that may be evaluated in this article, or claim that may be made by its manufacturer, is not guaranteed or endorsed by the publisher.
